# An immunotoxin targeting Ebola virus glycoprotein inhibits Ebola virus production from infected cells

**DOI:** 10.1371/journal.pone.0245024

**Published:** 2021-01-07

**Authors:** Yingyun Cai, Shuiqing Yu, Xiaoli Chi, Sheli R. Radoshitzky, Jens H. Kuhn, Edward A. Berger

**Affiliations:** 1 Integrated Research Facility at Fort Detrick, Division of Clinical Research, National Institute of Allergy and Infectious Diseases, National Institutes of Health, Fort Detrick, Frederick, Maryland, United States of America; 2 United States Army Medical Research Institute of Infectious Diseases, Fort Detrick, Frederick, Maryland, United States of America; 3 The Geneva Foundation, Tacoma, Washington, United States of America; 4 Laboratory of Viral Disease, National Institute of Allergy and Infectious Disease, National Institutes of Health, Bethesda, Maryland, United States of America; New York State Department of Health, UNITED STATES

## Abstract

Ebola virus (EBOV), a member of the mononegaviral family *Filoviridae*, causes severe disease associated with high lethality in humans. Despite enormous progress in development of EBOV medical countermeasures, no anti-EBOV treatment has been approved. We designed an immunotoxin in which a single-chain variable region fragment of the EBOV glycoprotein-specific monoclonal antibody 6D8 was fused to the effector domains of *Pseudomonas aeruginosa* exotoxin A (PE38). This immunotoxin, 6D8-PE38, bound specifically to cells expressing EBOV glycoproteins. Importantly, 6D8-PE38 targeted EBOV-infected cells, as evidenced by inhibition of infectious EBOV production from infected cells, including primary human macrophages. The data presented here provide a proof of concept for immunotoxin-based targeted killing of infected cells as a potential antiviral intervention for Ebola virus disease.

## Introduction

Ebola virus (EBOV; *Mononegavirales*: *Filoviridae*: *Ebolavirus*) causes Ebola virus disease (EVD), a severe human disease associated with lethality. There have been at least 19 EVD outbreaks since the discovery of EBOV in 1976 [1]. From 2013 to 2016, the largest EVD outbreak occurred in Western Africa (mainly Guinea, Liberia, and Sierra Leone), with 28,652 cases and 11, 325 deaths [2, 3]. The second-largest EVD outbreak occurred in Democratic Republic of the Congo from 2018 to 2020, with 3,481 reported cases and 2,299 deaths [4]. The high lethality associated with EVD (average ≈44% since 1976) underscores the need for effective medical countermeasures, such as vaccines and therapeutics, against EBOV. During and after 2013–2016 EVD outbreak, enormous progress was made in medical countermeasure development, resulting in United States (U.S.) Food and Drug Administration approval of the first vaccine, Ervebo, in December 2019 [5].

EBOV has a non-segmented, negative-sense, RNA genome and produces enveloped, filamentous virions. The EBOV genome is approximately 19 kb in length with seven genes in the following order: 3'-leader-*NP*-*VP35*-*VP40*-*GP*-*VP30*-*VP24*-*L*-trailer-5'. These seven genes encode seven structural proteins: nucleoprotein (NP) encapsidates the EBOV genomes and antigenomes; polymerase cofactor (VP35) mediates transcription/replication and immune evasion; matrix protein (VP40) drives virion assembly/egress, regulates transcription/replication, and mediates immune evasion; glycoprotein (GP_1,2_) mediates virion entry and inhibits the intrinsic immune response; transcriptional activator (VP30) mediates transcription initiation, re-initiation, enhancement, anti-termination, and *GP* mRNA editing and acts as an RNAi silencing suppressor; ribonucleoprotein complex-associated protein (VP24) regulates transcription/replication and virion assembly/egress, and large protein (L), which contains an RNA-directed RNA polymerase domain and mediates transcription/replication, mRNA maturation, and *GP* mRNA editing [6]. EBOV GP_1,2_ is synthesized as a preproprotein that is first cleaved by signalase during translocation into the endoplasmic reticulum to remove the signal peptide and then post-translationally cleaved by furin-like protease into GP_1_ and GP_2_ subunits that remain connected by a disulfide bond. The GP_1_-GP_2_ heterodimers trimerize to form mature GP_1,2_ peplomers that are incorporated into cellular membranes, including the plasma membrane, and ultimately into the virion envelope during virion budding [6]. Finally, co-transcription mRNA editing of the *GP* produces several secreted proteins (e.g., sGP, ssGP, Δ-peptide) with largely undetermined function [6].

Antibodies targeting EBOV GP_1,2_ are of great interest in various strategies for vaccine and therapeutic development. Although monoclonal antibody 114 (mAb114) and mAb cocktail REGN-EB3 were proven to be effective against EVD under certain conditions in the 2019 Pamoja Tulinde Maisha (PALM) randomized controlled clinical trial [7], lethality remains high, even in treated populations. The EBOV GP_1,2_-specific human mAb KZ52 demonstrated potent EBOV-neutralization activity *in vitro*, protected guinea pigs from disease caused by guinea-pig-adapted EBOV [8] but failed to protect EBOV-exposed nonhuman primates from developing lethal disease [9, 10]. On the other hand, EBOV non-neutralizing mAbs, such as 6D8, completely protected laboratory mice and rhesus monkeys when used in combination in a cocktail [11]. These and additional data indicate that the neutralization ability of an mAb does not solely predict its protective efficacy *in vivo* and other immune functions of an antibody, such as antibody-dependent cell-mediated cytotoxicity (ADCC), also play a role in protection [12].

We investigated an alternative application of mAb technology for direct targeted killing of EBOV-infected cells. Recombinant immunotoxins (RITs) are engineered chimeric proteins consisting of a cytotoxic protein moiety linked to a targeting protein moiety, such as an antibody variable domain (Fv) or a ligand that binds to a surface antigen selectively displayed on the target cell of interest. Most RITs in clinical trials or approved by the U.S. Food and Drug Administration contain a diphtheria toxin (DT), a *Pseudomonas* exotoxin A (PE), or a ricin cytotoxic moiety [13–15]. Wild-type PE consists of three domains: domain I is the cell-binding domain that targets low-density lipoprotein receptor-related protein 1 (LRP-1); domain II facilitates toxin translocation into the cytoplasm; and domain III is the catalytic domain that catalyzes the inactivation of eukaryotic translocation elongation factor 2 (EEF2) by ADP-ribosylation, thereby inhibiting protein synthesis and ultimately leading to cell death. A PE-based RIT typically contains the N-terminal-targeting moiety fused to a 38-kDa-truncated portion of PE (PE38), containing only domains II and III [16]. Therefore, in this study, we developed an RIT directly targeting EBOV GP_1,2_. We showed that this RIT selectively inhibits infectious EBOV production from infected cells, demonstrating the feasibility of RIT use as a novel antiviral EVD intervention.

## Materials and methods

### Cells

Human hepatocarcinoma Huh-7 cells were provided by Hideki Ebihara (Laboratory of Virology, National Institute of Allergy and Infectious Diseases, National Institutes of Health, Hamilton, Montana, United States of America [USA]). Grivet (*Chlorocebus aethiops*) kidney epithelial Vero E6 cells (#CRL-1568) were obtained from the American Type Culture Collection (Manassas, Virginia, USA). All cells were grown in Dulbecco’s modified Eagle medium (DMEM, Thermo Fisher Scientific, Waltham, Massachusetts [MA], USA) supplemented with 10% heat-inactivated fetal bovine serum (FBS, Millipore Sigma, St. Louis, Missouri, USA). Human monocyte-derived macrophages (MDMs) were generated from human whole blood (Biological Specificity Corporation, Colmar, Pennsylvania, USA), as described previously [17]. All cells were cultured at 37°C in a humidified 5% carbon dioxide atmosphere.

### Virus

Ebola virus/H.sapiens-tc/GIN/2014/Makona-C05 (GenBank #KP096420; hereafter: EBOV) was provided by the Public Health Agency of Canada (PHAC), Winnipeg, Canada. EBOV was propagated in Vero E6 cells at a multiplicity of infection (MOI) of 0.01 in DMEM supplemented with 2% FBS. Viral titers were quantified by plaque assay on Vero E6 cells, as described previously [18]. All experiments with EBOV were performed in a biosafety level 4 (BSL-4) laboratory of the Integrated Research Facility at Fort Detrick (IRF-Frederick) following approved standard operating procedures.

### Recombinant immunotoxin expression plasmid construction

**Fig 1:**
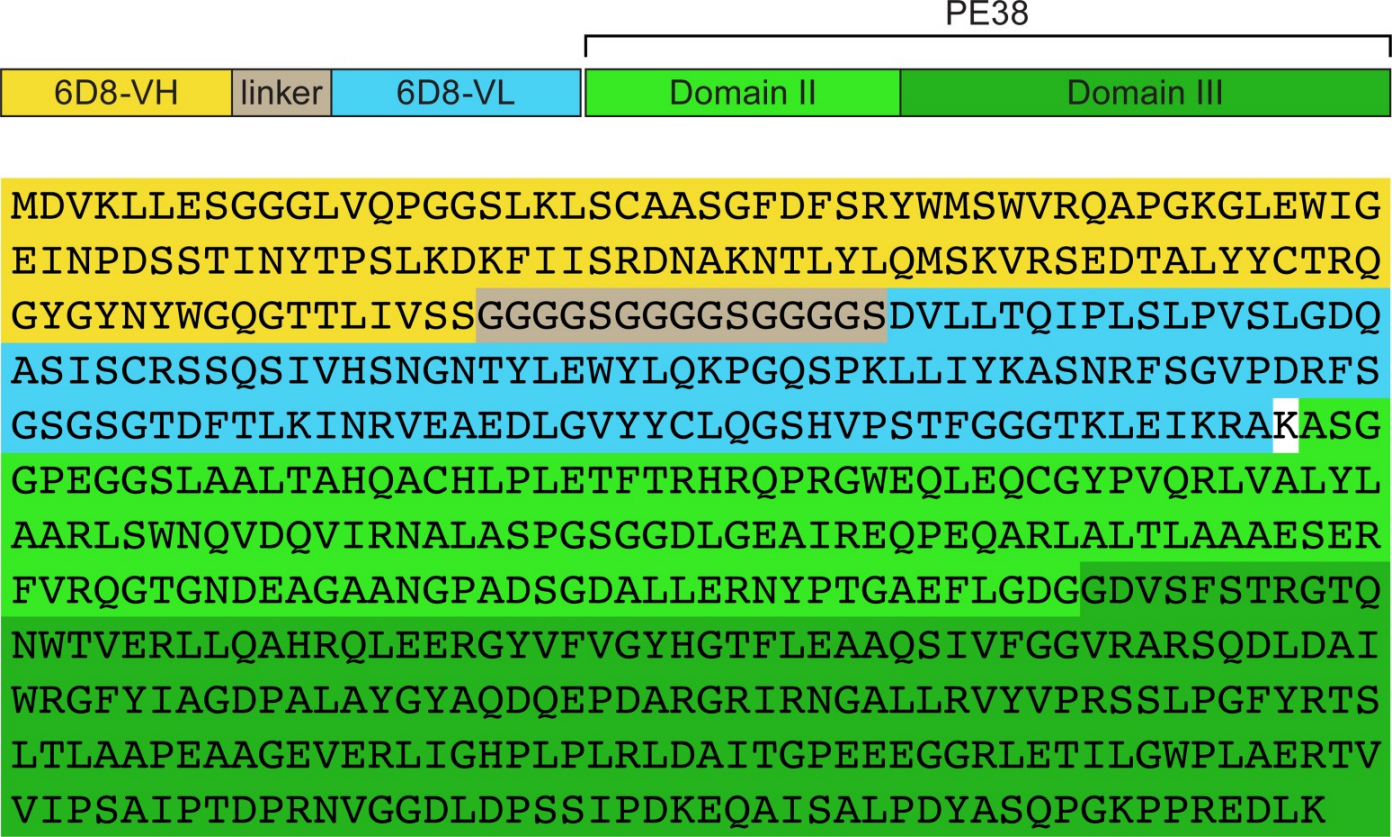
Schematic diagram and the amino acid sequence of the recombinant immunotoxin 6D8-PE38. The heavy chain of monoclonal antibody (mAb) 6D8 (6D8-VH, yellow) is connected to the light chain of mAb 6D8 (6D8-VL, blue) by a (Gly_4_Ser) linker (grey), which forms a single-chain variable fragment (scFV) of mAb 6D8. This scFv is fused to the effector domains II and III of *Pseudomonas* exotoxin A 38 (PE38, light and dark green, respectively).

The heavy-chain and light-chain sequences of mAb 6D8 were provided by John Dye (U.S. Army Medical Research Institute of Infectious Diseases, Fort Detrick, Maryland, USA). A single-chain variable fragment (scFv) of mAb 6D8—which contains the variable regions of the heavy (V_H_) and the light chains (V_L_) of mAb 6D8, connected by a 15-amino-acid linker (Gly_4_Ser)_3_ (Fig 1)—was generated by *de novo* synthesis and cloned into a pCR2.1 vector (ATUM, Newark, California [CA], USA) to generate pCR2.1-6D8scFv. pCR2.1-6D8scFv was digested with enzymes *Nde*I and *Hin*dIII. The resulting 6D8scFv fragment was used to replace the *Nde*I-*Hin*dIII fragment from PE-toxin expression plasmids pYC15-PE38 encoding YC15-PE38 [19]. The resulting 6D8-PE38 RIT expression plasmid was designated as p6D8-PE38.

### Recombinant immunotoxin expression and purification

6D8-PE38 RIT was expressed and purified, as described previously [19, 20]. Briefly, the RIT expression plasmid p6D8-PE38 was transformed into Max Efficiency DH5α *Escherichia coli* BL21(DE3) (New England Biolabs, Ipswich, MA, USA). Then, isopropyl-β-D-thiogalactopyranoside (IPTG, Millipore Sigma‎) was used to induce RIT expression. The inclusion body fraction was isolated from the bacterial pellets by lysozyme treatment and high-speed centrifugation (27,000 x*g* for 50 min at 4°C). The RIT was denatured and solubilized in denaturing buffer (6 M guanidine HCl, 2 mM EDTA, 100 mM Tris-HCl, pH 8), followed by reduction of disulfide bonds by addition of dithioerythritol powder (Millipore Sigma) to achieve a 10-mg/ml concentration, and incubation overnight at room temperature. The solubilized reduced RIT (MW = 66 kDa) was then refolded in refolding buffer (0.5 M arginine, 1 mM EDTA, 100 mM Tris-HCl, pH 9.5, 551 mg/ oxidized glutathione). The refolded proteins were dialyzed, and then purified by anion exchange chromatography using Q Sepharose and Mono Q anion exchangers (GE Healthcare Life Sciences, Pittsburgh, Pennsylvania, USA) and size-exclusion chromatography on a TSK3000 column (Millipore Sigma). The purified RIT concentration was determined using a bicinchoninic acid (BCA) protein assay, according to the manufacturer’s instructions (Thermo Fisher Scientific). The protein samples were separated by sodium dodecyl sulfate-polyacrylamide gel electrophoresis (SDS-PAGE) and stained with Coomassie blue solution (SimplyBlue Safe Stain, Thermo Fisher Scientific).

### Surface staining by flow cytometry

Vero E6 cells (4 x 10^5^ cells/well, 6-well plate format) were transiently transfected with 2.5 μg of empty or EBOV (variant Yambuku, isolate Mayinga) GP_1,2_ or Marburg virus (MARV, variant Mt. Elgon, isolate Musoke) GP_1,2_ pCAGGS expression plasmids [21, 22] using Roche X-tremeGENE HP DNA transfection reagent (Millipore Sigma). Cells were dissociated with enzyme-free cell-dissociation buffer (Thermo Fisher Scientific) 48 h post-transfection (p.t.) and stained with 2 μg/ml of mAb 6D8, followed by Alexa Fluor 488-conjugated goat anti-mouse immunoglobulin G (IgG) antibody (Thermo Fisher Scientific) on ice. As a control, all cells were stained with the same concentration of mouse IgG, followed by the same secondary antibody. These samples were analyzed with an LSRFortessa flow cytometer (Becton, Dickinson and Company, San Jose, CA, USA), and the data were analyzed with FlowJo software (Tree Star, Ashland, Oregon, USA). All experiments were performed in duplicates.

### Recombinant immunotoxin binding specificity analysis by flow cytometry

6D8-PE38 RIT was labeled with an Alexa Fluor 488 microscale protein labeling kit, according to the manufacturer’s instructions (Thermo Fisher Scientific). Vero E6 cells were transiently transfected with pCAGGS-EBOV-GP_1,2_ or control pCAGGS-MARV-GP_1,2_. 48 h p.t., cells were dissociated with enzyme-free cell-dissociation buffer. Transfected cells were washed with phosphate-buffered saline (Thermo Fisher Scientific) and then incubated with phosphate-buffered saline containing 3% bovine serum albumin (Millipore Sigma) at 4°C for 30 min to block non-specific binding. The cells were then incubated with different concentrations of labeled 6D8-PE38 RIT at 4°C for 1 h. The binding of immunotoxin on the cell surface was analyzed by flow cytometry. All experiments were performed in duplicates.

### Immunofluorescence assay

Huh-7 cells (4 x 10^4^ cells/well, 96-well plate format) or MDMs (1 x 10^5^ cells/well, 96-well plate format) were inoculated with EBOV at an MOI of 0.3 or 3. After 1 h of incubation at 37°C, viral inoculums were removed, and the cells were supplemented with DMEM containing 2% FBS. At various times post-inoculation, cell plates were fixed with 10% neutral buffered formalin (NBF, Thermo Fisher Scientific) for 24 h and then transferred from the BSL-4 to a BSL-2 laboratory. Without the permeabilization step, the plates were stained with mouse anti-EBOV GP_1,2_ mAb 6D8, followed by secondary Alexa Fluor 488-conjugated goat anti-mouse IgG antibody (Thermo Fisher Scientific). Cell nuclei were stained with Hoechst 33342 dye (Thermo Fisher Scientific). Fluorescent signal images were acquired with the Operetta high-content imaging system (PerkinElmer, Waltham, MA, USA). All experiments were performed in duplicates.

### Inhibition of infectious virus production assay

Huh-7 cells (4 x 10^4^ cells/well, 96-well plate format) or MDMs (1 x 10^5^ cells/well, 96-well plate format) were inoculated with EBOV at an MOI of 0.3 or 3. After 1 h of incubation at 37°C, viral inoculums were removed and the cells were treated with increasing concentrations of 6D8-PE38 RIT, control RIT YC15-PE38 [19], mAb 6D8 or control mouse IgG. At 48 h post-exposure (p.e.), tissue culture supernatants were collected. Virus titers in tissue culture supernatants were determined by plaque assay, as described previously [18]. All experiments were performed in duplicates.

### Data analysis

All statistical analyses were performed using GraphPad Prism 7 (GraphPad Software, San Diego, CA, USA). Statistically significant differences in viral titer were determined by unpaired Student t-test (*, *P* < 0.05, significant; **, *P* < 0.01, very significant; ***, *P* < 0.001, highly significant).

## Results

### Design and production of EBOV GP_1,2_-targeted recombinant immunotoxin 6D8-PE38

**Fig 2:**
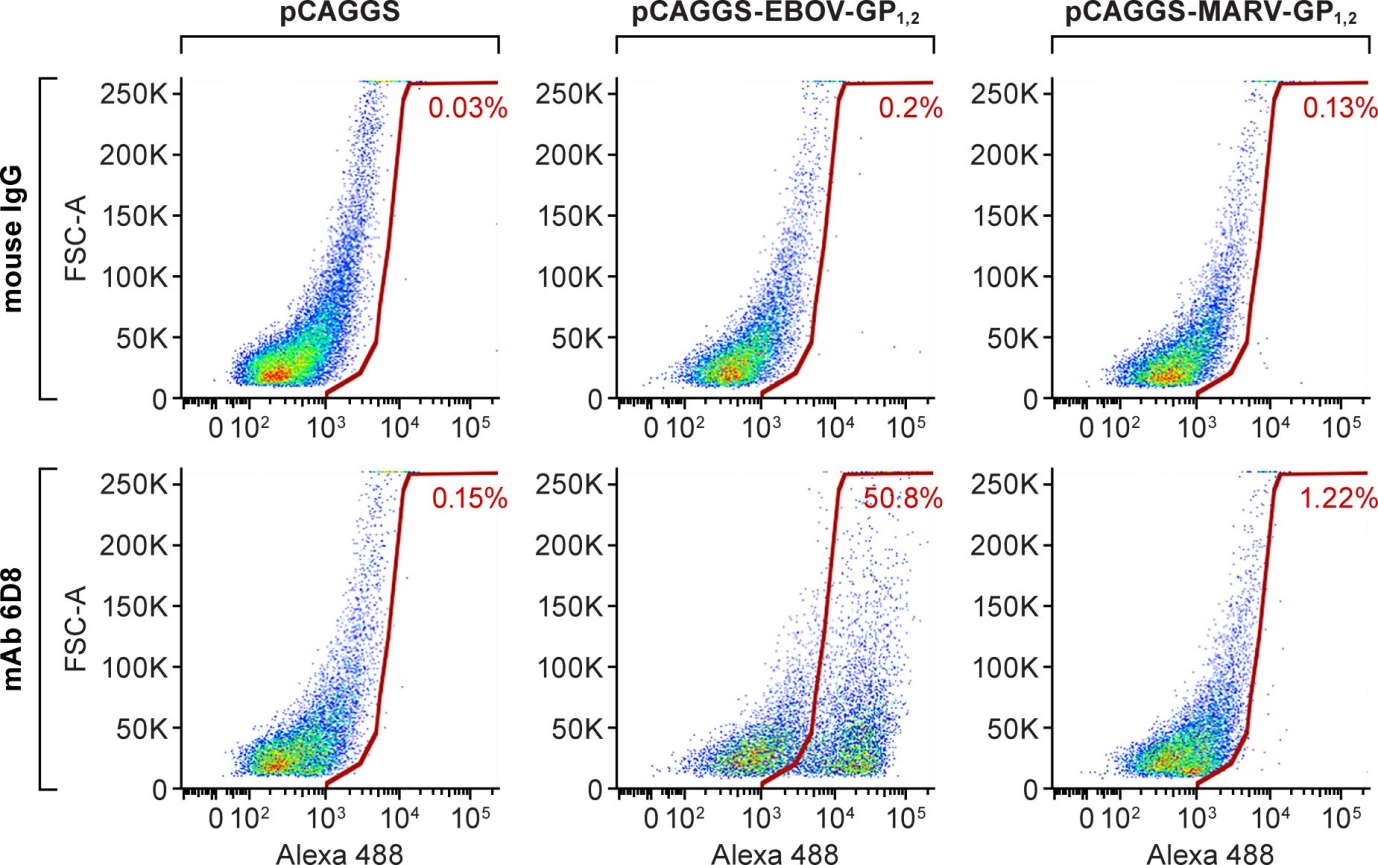
Recognition of EBOV GP_1,2_ expressed on the cell surface by mAb 6D8. Vero E6 cells were transfected with plasmids pCAGGS-EBOV-GP_1, 2_, pCAGGS-MARV-GP_1,2_, or pCAGGS. After 48 h, cells were dissociated with enzyme-free cell-dissociation buffer and stained with mAb 6D8 or control mouse immunoglobulin G (IgG), followed by Alexa Fluor 488-conjugated goat anti-mouse IgG antibody. FSC-A: forward scatter area.

To test the potential and feasibility of the immunotoxin as a therapeutic concept, we chose mAb 6D8 to design an immunotoxin targeting EBOV GP_1,2_. Developed by the U.S. Army Medical Research Institute of Infectious Diseases (USAMRIID), mAb 6D8 is a non-neutralizing mAb that recognizes a linear epitope in the mucin-like domain of GP_1, 2_’s GP_1_ subunit (amino acid residues 389–405: HNTPVYKLDISEATQVE; absent in GP_1_s of other ebolaviruses and filoviruses) [23]. mAb 6D8 specifically bound to the cells transfected with an EBOV GP_1,2_-expressing plasmid but not to cells transfected with a MARV GP_1,2_-expressing plasmid or empty vector control plasmid (Fig 2).

**Fig 3:**
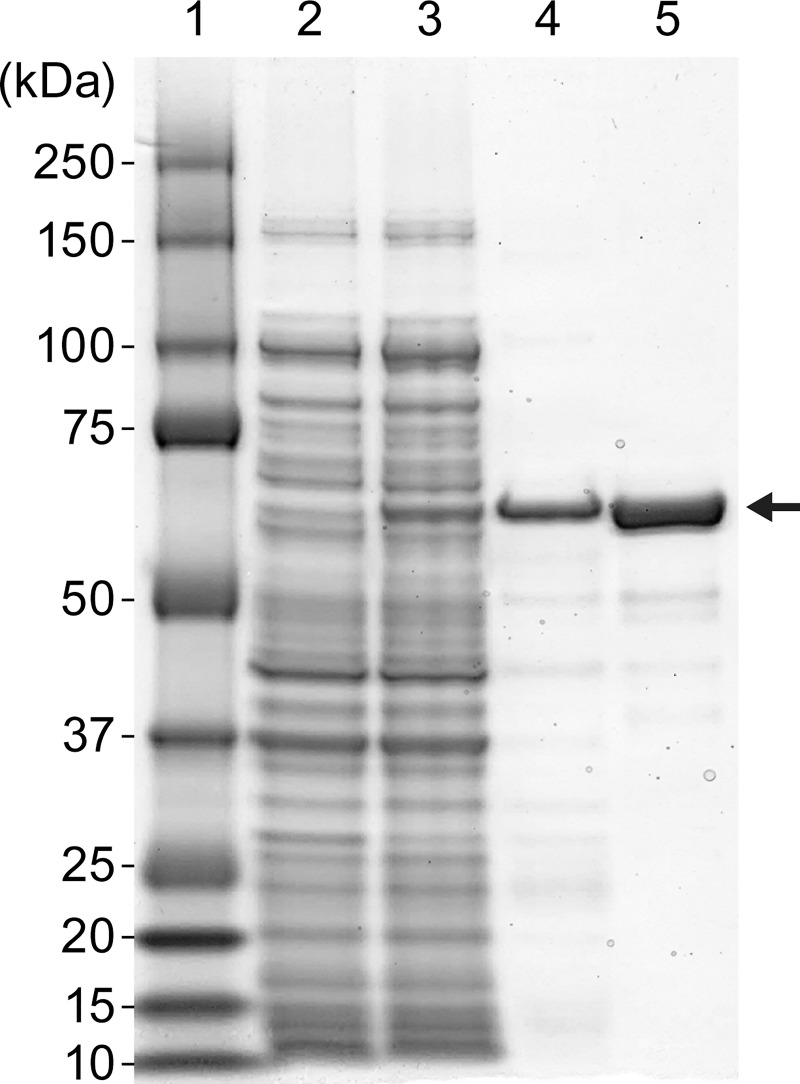
Expression and purification of 6D8-PE38 recombinant immunotoxin targeting the EBOV GP_1,2_. SDS-PAGE analysis of steps in purification of the 6D8-PE38 RIT. Lane 1: Molecular weight marker; Lane 2: Uninduced bacterial cell lysate; Lane 3: isopropyl-β-D-thiogalactopyranoside (IPTG)-induced bacterial cell lysate; Lane 4: Inclusion body preparation from induced cells; Lane 5: Purified 6D8-PE38 RIT.

An scFv of mAb 6D8, which contains the variable regions of the heavy and the light chains of mAb 6D8, connected by a 15-amino-acid linker (Gly_4_Ser)_3_, was linked to PE38, which contains only the translocation and effector domains of PE. We expressed this 6D8-PE38 RIT in *Escherichia coli* and purified it from the inclusion body fraction through standard protocols of solubilization, denaturation, refolding, ion exchange, and size-exclusion chromatography [24]. We detected the expression of the 6D8-PE38 RIT in bacterial culture upon IPTG induction and in the inclusion body fraction (Fig 3). The size and purity of the 6D8-PE38 RIT (MW = 66 kDa) was demonstrated by SDS-PAGE and Coomassie staining (Fig 3).

### Specific binding of 6D8-PE38 recombinant immunotoxin to cell surface-EBOV-GP

**Fig 4:**
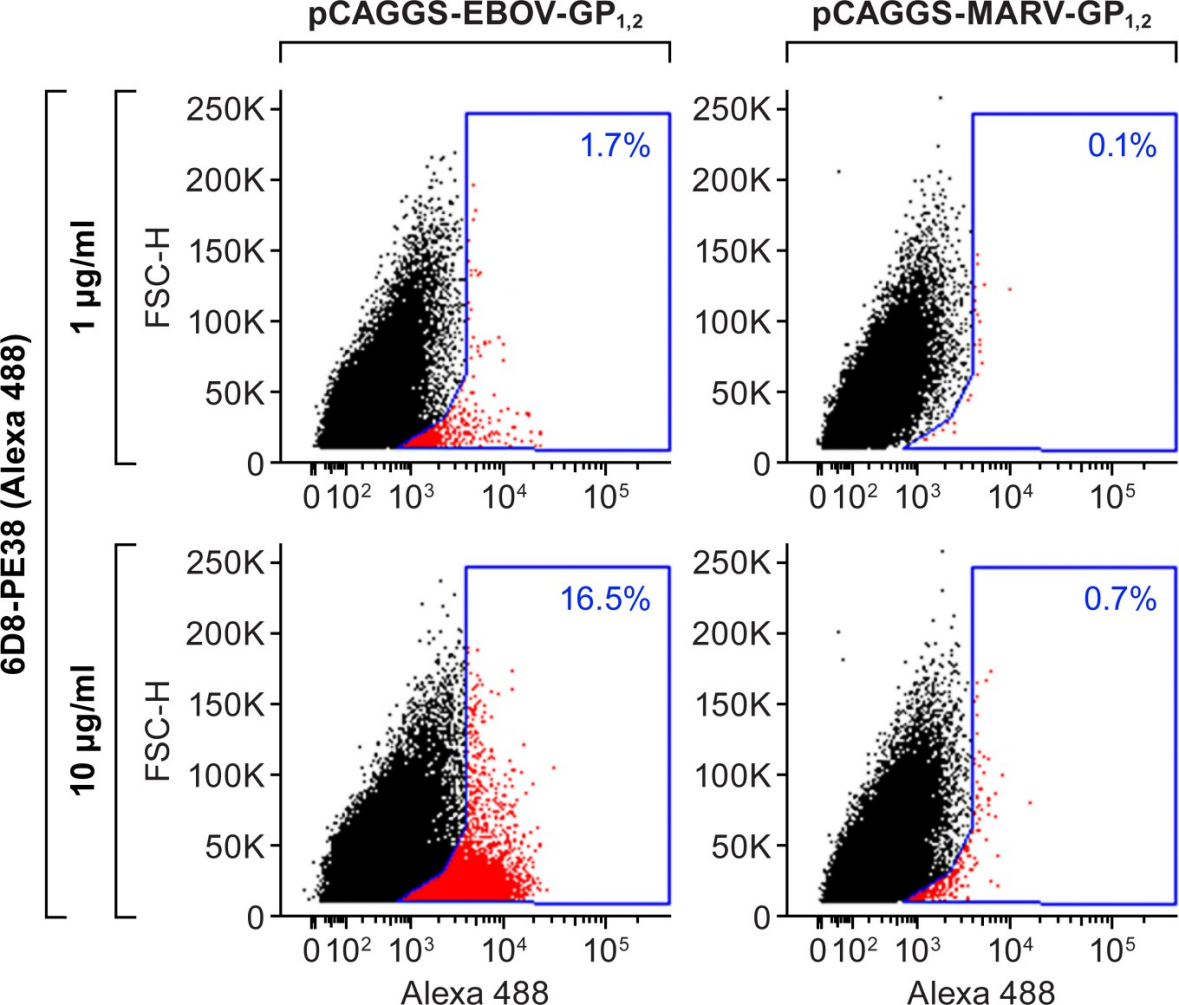
6D8-PE38 recombinant immunotoxin specially binds to EBOV GP_1,2_-expressing cells. 6D8-PE38 RIT was labeled with Alexa Fluor 488. Vero E6 cells were transfected with pCAGGS-EBOV-GP_1,2_ or control pCAGGS-MARV-GP_1,2_ plasmids. After 48 h, transfected cells were incubated with the indicated concentrations of labeled 6D8-PE38 RIT. Cell-bound RIT was detected by flow cytometry. FSC-H: forward scatter height.

The specific binding of the 6D8-PE38 RIT to EBOV GP_1,2_, expressed on the cell surface, was evaluated by flow cytometry. As shown in Fig 4, 6D8-PE38 RIT bound to EBOV GP_1,2_, expressed on transfected Vero E6 cells in a dose-dependent manner. Moreover, the RIT did not bind to the control cells, demonstrating the binding specificity of 6D8-PE38 RIT.

### Inhibition of infectious EBOV production by 6D8-PE38 recombinant immunotoxin

**Fig 5:**
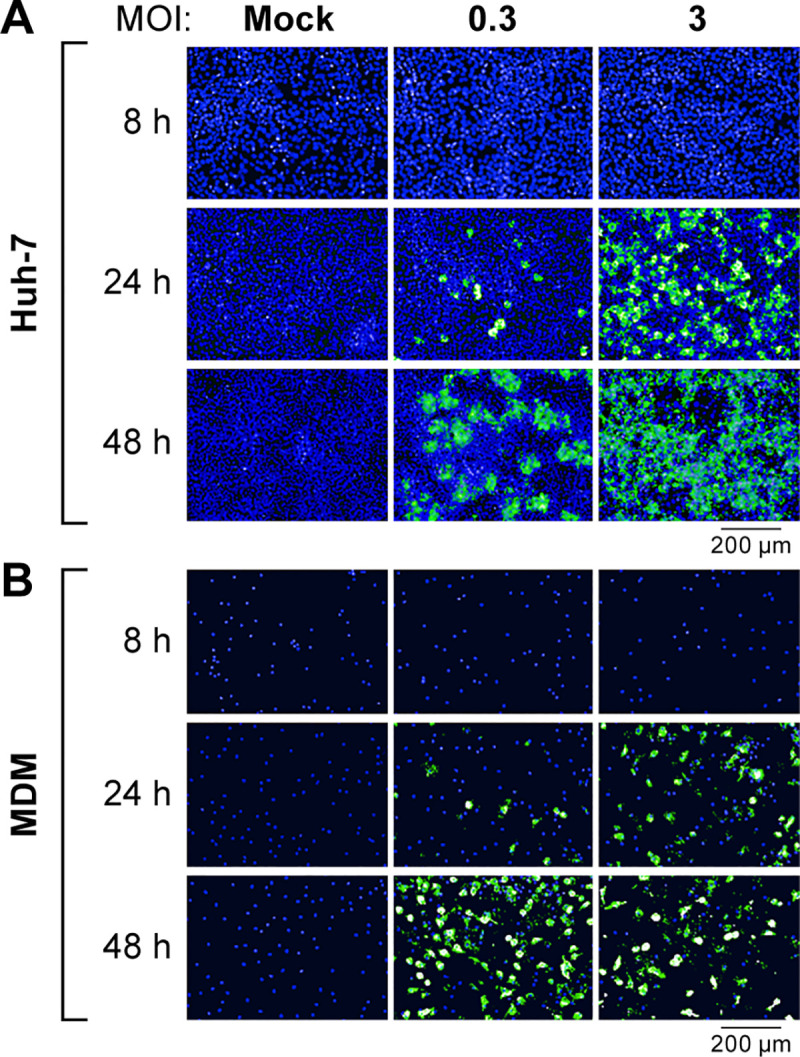
Surface staining with mAb 6D8 of EBOV-infected Huh-7 cells and MDMs. (A) Huh-7 cells or (B) MDMs were exposed to EBOV at an MOI of 0.3 or 3. At indicated times post-exposure, cells were stained with mAb 6D8. Fluorescence images were collected by high-content imaging.

To test the potential of the 6D8-PE38 RIT as a possible EBOV antiviral, we examined its effect on infectious virus production from cells infected with a wild-type EBOV obtained during the 2013–2016 EVD epidemic in Western Africa, caused by EBOV variant Makona [25, 26]. Since mAb 6D8 was generated with GP_1,2_ from the 1976 EBOV Yambuku variant as an immunogen [23], we first evaluated binding of mAb 6D8 to cells infected with EBOV variant Makona. Huh-7 cells or MDMs were exposed to live virus at an MOI of 0.3 or 3; mock-exposed cells served as negative controls. The expression of GP_1,2_ at 8, 24, and 48 h p.e. was assessed by staining with mAb 6D8 (Fig 5). GP_1,2_ expression was observed in both virus-exposed cell types in an MOI-dependent and time-dependent fashion. At 8 h p.e., GP_1,2_ expression was not detected in either cell type at either MOI; at 24 h p.e., GP_1,2_ expression was evident in both cell types, with higher levels at MOI 3; and at 48 h p.e., GP_1,2_ expression was robust in all cases. As expected, GP_1,2_ expression could not be detected in mock-infected cells.

**Fig 6:**
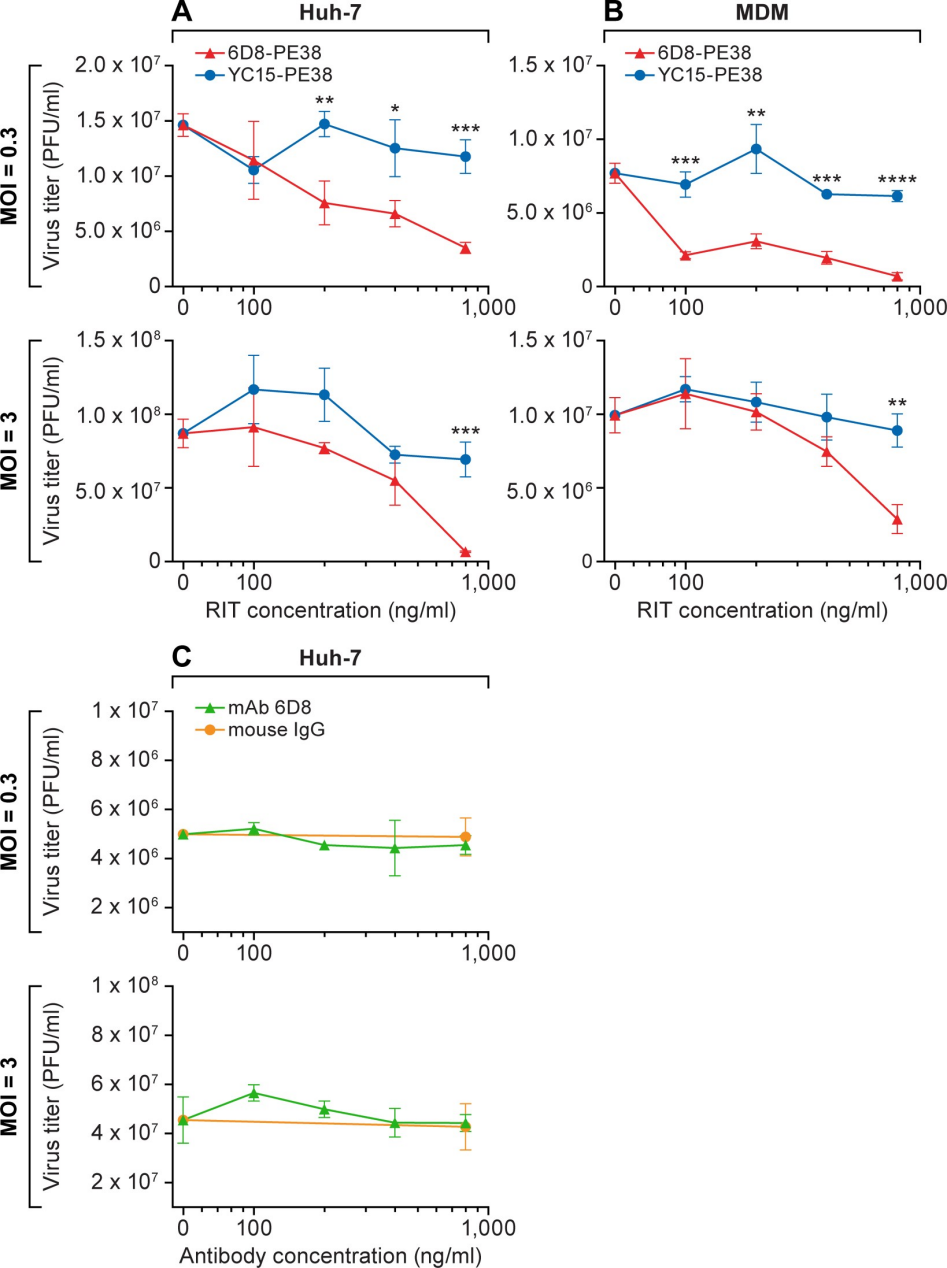
Effect of 6D8-PE38 recombinant immunotoxin on infectious EBOV production from infected cells. (A) Huh-7 cells and (B) MDMs were exposed to EBOV at the indicated MOIs. After 1 h, viral inoculums were removed, and the cells were treated with increasing concentrations of 6D8-PE38 RIT or control YC15-PE38 RIT. At 48 h post-exposure, tissue culture supernatants were collected, and virus titers were measured by plaque assay. Data represent the means ± the standard deviations of results from triplicate wells. *, *P* < 0.05; **, *P* < 0.01; ***, *P* < 0.001; ****, *P* < 0.0001 (Student t-test). (C) Huh-7 cells were exposed to EBOV at the indicated MOIs. After 1 h, viral inocula were removed, and the cells were treated with increasing concentrations of mAb 6D8 or control mouse IgG. At 48 h post-exposure, tissue culture supernatants were collected, and virus titers were measured by plaque assay. Data represent the means ± the standard deviations of results from triplicate wells.

With the confirmation of efficient mAb 6D8 binding to cells infected with EBOV, we next tested whether the 6D8-PE38 RIT was active against the infected cells. We used suppression of EBOV release as a readout of infected cell killing, as previously used in human immunodeficiency virus 1 (HIV-1) infection studies [27]. Huh-7 cells or MDMs were infected with EBOV at an MOI of 0.3 or 3. At 1 h p.e., the infected cells were washed to remove unbound virions and then treated with increasing concentrations of 6D8-PE38 RIT or control YC15-PE38 RIT. The amount of produced infectious virions was quantitated by plaque assay. We observed a dose-dependent reduction of infectious EBOV production from both cell types by 6D8-PE38 RIT, but not by the unrelated control YC15-PE38 RIT, in both Huh-7 cells (Fig 6A) and MDMs (Fig 6B). The observed potencies are comparable to reported data for other PE-based RITs with *in vivo* efficacy [28]. These effects cannot be explained by simple neutralization by the antibody component of the recombinant immunotoxin, since no reduction was observed with mAb 6D8 (Fig 6C), consistent with a previous report demonstrating the non-neutralizing effects of this antibody [23].

These data demonstrate the efficacy and specificity of the 6D8-PE38 RIT against EBOV infection.

## Discussion

The 2013–2016 EVD epidemic in Western Africa and recent outbreaks in the Democratic Republic of the Congo have highlighted the urgent need for effective antiviral therapeutics to meet future outbreaks. A number of investigational agents with promising activities *in vitro* and in animal models have been administered in the field and advanced to clinical studies [7, 29, 30]. Particularly promising are specific mAbs directed against EBOV GP_1,2_ [31, 32], used singly (e.g., mAb114) or in combination (e.g., REGN-EB3 mAb cocktail); the therapeutic potential of these antibodies has been the focus of several clinical trials [7, 33, 34].

This study is a first step toward an alternative EVD treatment strategy, using anti-EBOV antibodies for direct killing of virus target cells. Antibody-based target cell killing has been advanced in cancer treatment and is moving to the forefront of therapeutic approaches against diverse viral pathogens, including HIV-1. Specific efforts include adoptive transfer of T cells, e.g., natural virus-specific T cells expanded *ex vivo* [35] or genetically modified T cells expressing cloned T-cell receptors or chimeric antigen receptors [36, 37]; another T-cell-mediated technology involves bispecific T-cell-engaging antibodies [38]. Distinct from these technically complex cell-mediated strategies, RIT technology typically involves intravenous infusion of a purified protein for direct killing of infected cells [39, 40]; RITs targeting HIV-1 and other specific viral pathogens have been described [41–43]. We propose that immunotoxins, such as the 6D8-PE38 RIT reported herein, have potential therapeutic utility against EBOV. In this context, reported features of the original mAb 6D8 are noteworthy. This antibody strongly protected EBOV-exposed laboratory animals despite the absence of direct neutralizing activity; however, neutralization in the presence of complement was observed, suggesting that the *in vivo* protection reflected targeted killing of infected cells by either complement-mediated lysis or ADCC [23]. Indeed, Fc-mediated activities have been observed for many EBOV-protective antibodies [12, 44], including components of leading therapeutic cocktails, such as REGN-EB3 [45]. RIT technology, which is based on replacing the Fc domain of an antibody with a cytotoxic protein moiety, offers a direct approach for targeted killing of EBOV-infected cells, and our results indicate the efficacy and specificity of such an RIT against EBOV infection. It should be noted that the observed suppressive activity of the 6d8-PE38 RIT cannot be explained by neutralization, since mAb 6D8 is a non-neutralization antibody [23] and because the 6D8-PE38 RIT was added 1 h p.e., after removal of excess virus. A significant advantage of the 6D8-PE38 RIT, compared to the original mAb 6D8, is that the required dosage to achieve efficacy likely will be orders of magnitude lower. The *in vivo* efficacy range for a PE-based RIT is typically 0.05 mg/kg or less per dose [46], as contrasted with the 5–50 mg/kg range for mAb 6D8 [23] as well as other individual mAbs in efficacious anti-EBOV cocktails [7, 33, 34, 45, 47]. The mechanism of action of the 6D8-PE38 RIT tested here, which our data strongly suggest being preferential killing of EBOV-infected, will have to be evaluated further. Since EBOV, or over-expressed EBOV GP_1,2_, causes severe cytopathic effects in infected/transfected cells [48–50], we could not separate these effects from cytopathic effects induced by the RIT. It is thus formally possible that the RIT exerted its EBOV-inhibitory effect by other or additional means, for instance via steric hindrance caused by the cytotoxic (here, PE38) moiety. Such alternative or additional actions may also contribute to off-target effects of the RIT on various cell types that will have to be defined.

Cell-culture studies have revealed synergistic activities between particular combinations of EBOV-inhibiting drugs [18]. In considering the RIT, relevance was based on previous cell-culture studies with HIV-1 that demonstrated robust synergy between one agent that inhibits virus replication and another that directly kills infected cells. For instance, combination treatment with an HIV-1 reverse transcriptase inhibitor (azidothymidine [AZT] or didanosine [DDI]) plus an RIT (CD4-PE40) completely eliminated HIV-1 from cultures, a result not approached with either agent alone [51]. Similar cooperative activities were observed in a humanized mouse model; combination treatment with HIV-1 replication blockers (reverse transcriptase and protease inhibitors) plus RIT CD4-PE40 nearly completely prevented virus rebound after cessation of treatment, in marked contrast with the limited effect of either class of agents alone [51].

We hypothesize that RITs, similar to the proof-of-principle 6D8-PE38 RIT described here, will robustly complement EBOV-replication inhibitors. For example, an RIT might significantly enhance the therapeutic efficacy of a small-molecule RNA synthesis inhibitor, such as remdesivir, beyond what was observed clinically with remdesivir alone [7]. The molecular weights of mAbs and RITs are too large to pass through selectively permeable endothelial cell borders (e.g., blood-brain or blood-testis barriers). Hence, mAbs and RITs could only be used therapeutically to address acute EVD. However, in combination with EBOV-specific small-molecule antivirals that cross such barriers, acute and persistent EBOV infection in immune-privileged sites of EVD survivors [52] could be addressed simultaneously and synergistically [53]. In addition, recent studies suggest that RITs could be modified to enhance entry in privileged sites [54]. RITs could also be combined with anti-EBOV mAbs that act primarily by direct neutralization; these replication-blocking activities might be significantly complemented by the direct targeting cell-killing activity of the RIT. To achieve such a cooperative effect, mAb selection must avoid binding competition between the two antibody-based agents. mAb 6D8 binds to a linear epitope in the mucin-like domain of EBOV GP_1,2_ [23, 55], which is physically separate from the GP_1,2_ core, base, and glycan cap that harbors the binding sites for the potent neutralizing mAbs of current therapeutic interest [12, 45, 55–57]. In recent years, numerous EBOV-specific mAbs have been described with a broad variety of properties [12] that could be taken into account for the development of EBOV-specific RITs with improved affinity and/or more preferable GP_1,2_-binding sites. In addition, mAbs could be chosen that bind the GP_1,2_s of multiple ebolaviruses (e.g., those of Bundibugyo virus [BDBV], Sudan virus [SUDV], and/or Taï Forest virus [TAFV]), or even bind the GP_1,2_s of non-ebolavirus filoviruses, thereby resulting in broad-spectrum RITs.

Finally, we must consider general clinical concerns that have arisen during the extensive studies with anti-cancer RITs [39, 40]. The toxin moieties of clinically advanced RITs are generally derived from bacterial toxins. The immunogenicity of these nonhuman components has been a critical obstacle, prompting extensive de-immunization efforts through genetic modification [58]. This concern is minimized for EVD by the acute nature of the disease and the resulting short treatment window. Another obstacle for RIT therapy is the potential for dose-limiting adverse effects, including on-target toxicity against normal cells expressing the same target molecule and off-target systemic toxicity, particularly vascular leak syndrome associated with toxin-induced damage to endothelial cells. On-target toxicity would likely be minimal for the 6D8-PE38 RIT, since surface expression of the target antigen is limited to EBOV-infected cells. The extent of off-target toxicity varies amongst different RITs, and can be reduced by specific genetic modifications of the toxin domain as has been reported for several PE-based RITs [59–61]. We hypothesize that none of these obstacles is insurmountable and that proof-of-principle *in vivo* experiments ought to be performed to further evaluate the therapeutic potential of RITs against EVD.

## Supporting information

S1 Raw image (TIF)

## References

[1] Jacob ST, Crozier I, Fischer WA II, Hewlett A, Kraft CS, Vega M-A, et al Ebola virus disease. Nat Rev Dis Primers. 2020;6(1):13 Epub 2020/02/23. 10.1038/s41572-020-0147-3 32080199 PMC7223853

[2] World Health Organization. Ebola outbreak 2014–2015. http://www.who.int/csr/disease/ebola/en/. 2017.

[3] Bullard SG. A day-by-day chronicle of the 2013–2016 Ebola outbreak. Cham, Switzerland: Springer; 2018.

[4] World Health Organization. Ebola in the Democratic Republic of the Congo, North Kivu, Ituri 2018–2020. https://www.who.int/emergencies/diseases/ebola/drc-2019. 2020.

[5] Food and Drug Administration. First FDA-approved vaccine for the prevention of Ebola virus disease, marking a critical milestone in public health preparedness and response. https://www.fda.gov/news-events/press-announcements/first-fda-approved-vaccine-prevention-ebola-virus-disease-marking-critical-milestone-public-health. 2019.

[6] Kuhn JH, Amarasinghe GK, Perry DL. *Filoviridae*. In: Howley PM, Knipe DM, Whelan SPJ, editors. Fields Virology. 1 (Emerging Viruses). 7th ed Philadelphia, USA: Wolters Kluwer/Lippincott Williams & Wilkins; 2020 p. 449–503.

[7] Mulangu S, Dodd LE, Davey RT Jr., Tshiani Mbaya O, Proschan M, Mukadi D, et al A randomized, controlled trial of Ebola virus disease therapeutics. N Engl J Med. 2019;381(24):2293–303. Epub 2019/11/28. 10.1056/NEJMoa1910993 . 31774950 PMC10680050

[8] Parren PWHI, Geisbert TW, Maruyama T, Jahrling PB, Burton DR. Pre- and postexposure prophylaxis of Ebola virus infection in an animal model by passive transfer of a neutralizing human antibody. J Virol. 2002;76(12):6408–12. Epub 2002/05/22. 10.1128/jvi.76.12.6408-6412.2002 12021376 PMC136210

[9] Maruyama T, Rodriguez LL, Jahrling PB, Sanchez A, Khan AS, Nichol ST, et al Ebola virus can be effectively neutralized by antibody produced in natural human infection. J Virol. 1999;73(7):6024–30. Epub 1999/06/11. 10.1128/JVI.73.7.6024-6030.1999 10364354 PMC112663

[10] Oswald WB, Geisbert TW, Davis KJ, Geisbert JB, Sullivan NJ, Jahrling PB, et al Neutralizing antibody fails to impact the course of Ebola virus infection in monkeys. PLoS Pathog. 2007;3(1):e9 Epub 2007/01/24. 10.1371/journal.ppat.0030009 17238286 PMC1779296

[11] Pettitt J, Zeitlin L, Kim DH, Working C, Johnson JC, Bohorov O, et al Therapeutic intervention of Ebola virus infection in rhesus macaques with the MB-003 monoclonal antibody cocktail. Sci Transl Med. 2013;5(199):199ra13 Epub 2013/08/24. 10.1126/scitranslmed.3006608 . 23966302

[12] Saphire EO, Schendel SL, Fusco ML, Gangavarapu K, Gunn BM, Wec AZ, et al Systematic analysis of monoclonal antibodies against Ebola virus GP defines features that contribute to protection. Cell. 2018;174(4):938–52.e13. Epub 2018/08/11. 10.1016/j.cell.2018.07.033 30096313 PMC6102396

[13] Shafiee F, Aucoin MG, Jahanian-Najafabadi A. Targeted diphtheria toxin-based therapy: a review article. Front Microbiol. 2019;10:2340 Epub 2019/11/05. 10.3389/fmicb.2019.02340 31681205 PMC6813239

[14] Abou Dalle I, Ravandi F. Moxetumomab pasudotox for the treatment of relapsed and/or refractory hairy cell leukemia. Expert Rev Hematol. 2019;12(9):707–14. Epub 2019/07/13. 10.1080/17474086.2019.1643231 . 31298972

[15] Polito L, Djemil A, Bortolotti M. Plant toxin-based immunotoxins for cancer therapy: a short overview. Biomedicines. 2016;4(2):12 Epub 2017/05/26. 10.3390/biomedicines4020012 28536379 PMC5344252

[16] Weldon JE, Pastan I. A guide to taming a toxin—recombinant immunotoxins constructed from *Pseudomonas* exotoxin A for the treatment of cancer. FEBS J. 2011;278(23):4683–700. Epub 2011/05/19. 10.1111/j.1742-4658.2011.08182.x 21585657 PMC3179548

[17] Cong Y, McArthur MA, Cohen M, Jahrling PB, Janosko KB, Josleyn N, et al Characterization of yellow fever virus infection of human and non-human primate antigen presenting cells and their interaction with CD4^+^ T cells. PLoS Negl Trop Dis. 2016;10(5):e0004709 Epub 2016/05/19. 10.1371/journal.pntd.0004709 27191161 PMC4871483

[18] Dyall J, Nelson EA, DeWald LE, Guha R, Hart BJ, Zhou H, et al Identification of combinations of approved drugs with synergistic activity against Ebola virus in cell cultures. J Infect Dis. 2018;218 Suppl 5:S672–S8. Epub 2018/06/26. 10.1093/infdis/jiy304 29939303 PMC6249579

[19] Cai Y, Berger EA. An immunotoxin targeting the gH glycoprotein of KSHV for selective killing of cells in the lytic phase of infection. Antiviral Res. 2011;90(3):143–50. Epub 2011/03/29. 10.1016/j.antiviral.2011.03.175 21440007 PMC3102131

[20] Chatterjee D, Chandran B, Berger EA. Selective killing of Kaposi's sarcoma-associated herpesvirus lytically infected cells with a recombinant immunotoxin targeting the viral gpK8.1A envelope glycoprotein. MAbs. 2012;4(2):233–42. Epub 2012/03/02. 10.4161/mabs.4.2.19262 22377676 PMC3361659

[21] Wahl-Jensen VM, Afanasieva TA, Seebach J, Ströher U, Feldmann H, Schnittler H-J. Effects of Ebola virus glycoproteins on endothelial cell activation and barrier function. J Virol. 2005;79(16):10442–50. Epub 2005/07/30. 10.1128/JVI.79.16.10442-10450.2005 16051836 PMC1182673

[22] Huang IC, Bailey CC, Weyer JL, Radoshitzky SR, Becker MM, Chiang JJ, et al Distinct patterns of IFITM-mediated restriction of filoviruses, SARS coronavirus, and influenza A virus. PLoS Pathog. 2011;7(1):e1001258 Epub 2011/01/22. 10.1371/journal.ppat.1001258 21253575 PMC3017121

[23] Wilson JA, Hevey M, Bakken R, Guest S, Bray M, Schmaljohn AL, et al Epitopes involved in antibody-mediated protection from Ebola virus. Science. 2000;287(5458):1664–6. Epub 2000/03/04. 10.1126/science.287.5458.1664 . 10698744

[24] Pastan R, Beers TK, Bera I. Recombinant immunotoxins in the treatment of cancer. In: Lo BKC, editor. Methods in Molecular Biology. Totowa, NJ, USA: Humana Press; 2003 p. 503–18. 10.1385/1-59259-666-5:503 14970517

[25] Baize S, Pannetier D, Oestereich L, Rieger T, Koivogui L, Magassouba NF, et al Emergence of Zaire Ebola virus disease in Guinea. N Engl J Med. 2014;371(15):1418–25. Epub 2014/04/18. 10.1056/NEJMoa1404505 . 24738640

[26] Kuhn JH, Andersen KG, Baize S, Bào Y, Bavari S, Berthet N, et al Nomenclature- and database-compatible names for the two Ebola virus variants that emerged in Guinea and the Democratic Republic of the Congo in 2014. Viruses. 2014;6(11):4760–99. Epub 2014/11/24. 10.3390/v6114760 25421896 PMC4246247

[27] Ashorn P, Moss B, Berger EA. Anti-HIV effects of CD4- *Pseudomonas* exotoxin on human lymphocyte and monocyte/macrophage cell lines. Ann N Y Acad Sci. 1990;616:149–54. Epub 1990/01/01. 10.1111/j.1749-6632.1990.tb17835.x . 2078015

[28] Kreitman RJ, Margulies I, Stetler-Stevenson M, Wang Q-C, FitzGerald DJP, Pastan I. Cytotoxic activity of disulfide-stabilized recombinant immunotoxin RFB4(dsFv)-PE38 (BL22) toward fresh malignant cells from patients with B-cell leukemias. Clin Cancer Res. 2000;6(4):1476–87. Epub 2000/04/25. . 10778980

[29] Davey RT Jr., Dodd L, Proschan M, Jahrling P, Hensley L, Higgs E, et al The past need not be prologue: recommendations for testing and positioning the most-promising medical countermeasures for the next outbreak of Ebola virus infection. J Infect Dis. 2018;218 Suppl 5:S690–S7. Epub 2018/07/23. 10.1093/infdis/jiy334 30032267 PMC6249585

[30] Hoenen T, Groseth A, Feldmann H. Therapeutic strategies to target the Ebola virus life cycle. Nat Rev Microbiol. 2019;17(10):593–606. Epub 2019/07/26. 10.1038/s41579-019-0233-2 . 31341272

[31] Zeitlin L, Whaley KJ, Olinger GG, Jacobs M, Gopal R, Qiu X, et al Antibody therapeutics for Ebola virus disease. Curr Opin Virol. 2016;17:45–9. Epub 2016/01/31. 10.1016/j.coviro.2016.01.006 26826442 PMC4902774

[32] Saphire EO, Schendel SL, Gunn BM, Milligan JC, Alter G. Antibody-mediated protection against Ebola virus. Nat Immunol. 2018;19(11):1169–78. Epub 2018/10/20. 10.1038/s41590-018-0233-9 30333617 PMC6814399

[33] Sivapalasingam S, Kamal M, Slim R, Hosain R, Shao W, Stoltz R, et al Safety, pharmacokinetics, and immunogenicity of a co-formulated cocktail of three human monoclonal antibodies targeting Ebola virus glycoprotein in healthy adults: a randomised, first-in-human phase 1 study. Lancet Infect Dis. 2018;18(8):884–93. Epub 2018/06/23. 10.1016/S1473-3099(18)30397-9 . 29929783

[34] Gaudinski MR, Coates EE, Novik L, Widge A, Houser KV, Burch E, et al Safety, tolerability, pharmacokinetics, and immunogenicity of the therapeutic monoclonal antibody mAb114 targeting Ebola virus glycoprotein (VRC 608): an open-label phase 1 study. Lancet. 2019;393(10174):889–98. Epub 2019/01/29. 10.1016/S0140-6736(19)30036-4 30686586 PMC6436835

[35] Barrett AJ, Prockop S, Bollard CM. Virus-specific T cells: broadening applicability. Biol Blood Marrow Transplant. 2018;24(1):13–8. Epub 2017/10/17. 10.1016/j.bbmt.2017.10.004 29032062 PMC5743764

[36] June CH, Levine BL. T cell engineering as therapy for cancer and HIV: our synthetic future. Philos Trans R Soc Lond B Biol Sci. 2015;370(1680):20140374 Epub 2015/09/30. 10.1098/rstb.2014.0374 26416683 PMC4634001

[37] Wagner TA. Quarter century of anti-HIV CAR T cells. Curr HIV/AIDS Rep. 2018;15(2):147–54. Epub 2018/03/04. 10.1007/s11904-018-0388-x 29500712 PMC5884727

[38] Fabozzi G, Pegu A, Koup RA, Petrovas C. Bispecific antibodies: potential immunotherapies for HIV treatment. Methods. 2019;154:118–24. Epub 2018/10/24. 10.1016/j.ymeth.2018.10.010 30352254 PMC6348037

[39] Alewine C, Hassan R, Pastan I. Advances in anticancer immunotoxin therapy. Oncologist. 2015;20(2):176–85. Epub 2015/01/07. 10.1634/theoncologist.2014-0358 25561510 PMC4319635

[40] Kim J-S, Jun S-Y, Kim Y-S. Critical issues in the development of immunotoxins for anticancer therapy. J Pharm Sci. 2020;109(1):104–15. Epub 2019/11/02. 10.1016/j.xphs.2019.10.037 . 31669121

[41] Pincus SH, Fang H, Wilkinson R. Anti-HIV immunotoxins. In: Muzykantov VR, Torchilin VP, editors. Biomedical Aspects of Drug Targeting. Norwell, USA: Kluwer Academic Publishers; 2003 p. 403–17.

[42] Berger EA, Pastan I. Immunotoxin complementation of HAART to deplete persisting HIV-infected cell reservoirs. PLoS Pathog. 2010;6(6):e1000803 Epub 2010/06/16. 10.1371/journal.ppat.1000803 20548940 PMC2883583

[43] Spiess K, Jakobsen MH, Kledal TN, Rosenkilde MM. The future of antiviral immunotoxins. J Leukoc Biol. 2016;99(6):911–25. Epub 2016/01/06. 10.1189/jlb.2MR1015-468R . 26729815

[44] King LB, West BR, Schendel SL, Saphire EO. The structural basis for filovirus neutralization by monoclonal antibodies. Curr Opin Immunol. 2018;53:196–202. Epub 2018/06/26. 10.1016/j.coi.2018.05.001 29940415 PMC6141344

[45] Pascal KE, Dudgeon D, Trefry JC, Anantpadma M, Sakurai Y, Murin CD, et al Development of clinical-stage human monoclonal antibodies that treat advanced Ebola virus disease in nonhuman primates. J Infect Dis. 2018;218 Suppl 5:S612–S26. Epub 2018/06/04. 10.1093/infdis/jiy285 29860496 PMC6249601

[46] Kreitman RJ, Dearden C, Zinzani PL, Delgado J, Karlin L, Robak T, et al Moxetumomab pasudotox in relapsed/refractory hairy cell leukemia. Leukemia. 2018;32(8):1768–77. Epub 2018/07/22. 10.1038/s41375-018-0210-1 30030507 PMC6087717

[47] Qiu X, Wong G, Audet J, Bello A, Fernando L, Alimonti JB, et al Reversion of advanced Ebola virus disease in nonhuman primates with ZMapp. Nature. 2014;514(7520):47–53. Epub 2014/08/30. 10.1038/nature13777 25171469 PMC4214273

[48] Groseth A, Marzi A, Hoenen T, Herwig A, Gardner D, Becker S, et al The Ebola virus glycoprotein contributes to but is not sufficient for virulence in vivo. PLoS Pathog. 2012;8(8):e1002847 Epub 2012/08/10. 10.1371/journal.ppat.1002847 22876185 PMC3410889

[49] Takada A, Watanabe S, Ito H, Okazaki K, Kida H, Kawaoka Y. Downregulation of β1 integrins by Ebola virus glycoprotein: implication for virus entry. Virology. 2000;278(1):20–6. Epub 2000/12/09. 10.1006/viro.2000.0601 . 11112476

[50] Simmons G, Wool-Lewis RJ, Baribaud F, Netter RC, Bates P. Ebola virus glycoproteins induce global surface protein down-modulation and loss of cell adherence. J Virol. 2002;76(5):2518–28. Epub 2002/02/12. 10.1128/jvi.76.5.2518-2528.2002 11836430 PMC153797

[51] Ashorn P, Moss B, Weinstein JN, Chaudhary VK, FitzGerald DJ, Pastan I, et al Elimination of infectious human immunodeficiency virus from human T-cell cultures by synergistic action of CD4-*Pseudomonas* exotoxin and reverse transcriptase inhibitors. Proc Natl Acad Sci U S A. 1990;87(22):8889–93. Epub 1990/11/01. 10.1073/pnas.87.22.8889 1701055 PMC55065

[52] Caviness K, Kuhn JH, Palacios G. Ebola virus persistence as a new focus in clinical research. Curr Opin Virol. 2017;23:43–8. Epub 2017/03/25. 10.1016/j.coviro.2017.02.006 . 28340374

[53] Iversen PL, Kane CD, Zeng X, Panchal RG, Warren TK, Radoshitzky SR, et al Recent successes in therapeutics for Ebola virus disease: no time for complacency. Lancet Infect Dis. 2020;20(9):e231–e7. Epub 2020/06/22. 10.1016/S1473-3099(20)30282-6 32563280 PMC7302789

[54] Beilhartz GL, Sugiman-Marangos SN, Melnyk RA. Repurposing bacterial toxins for intracellular delivery of therapeutic proteins. Biochem Pharmacol. 2017;142:13–20. Epub 2017/04/15. 10.1016/j.bcp.2017.04.009 . 28408344

[55] Davidson E, Bryan C, Fong RH, Barnes T, Pfaff JM, Mabila M, et al Mechanism of binding to Ebola virus glycoprotein by the ZMapp, ZMAb, and MB-003 cocktail antibodies. J Virol. 2015;89(21):10982–92. Epub 2015/08/28. 10.1128/JVI.01490-15 26311869 PMC4621129

[56] Murin CD, Fusco ML, Bornholdt ZA, Qiu X, Olinger GG, Zeitlin L, et al Structures of protective antibodies reveal sites of vulnerability on Ebola virus. Proc Natl Acad Sci U S A. 2014;111(48):17182–7. Epub 2014/11/19. 10.1073/pnas.1414164111 25404321 PMC4260551

[57] Misasi J, Gilman MSA, Kanekiyo M, Gui M, Cagigi A, Mulangu S, et al Structural and molecular basis for Ebola virus neutralization by protective human antibodies. Science. 2016;351(6279):1343–6. Epub 2016/02/27. 10.1126/science.aad6117 26917592 PMC5241105

[58] Mazor R, King EM, Pastan I. Strategies to reduce the immunogenicity of recombinant immunotoxins. Am J Pathol. 2018;188(8):1736–43. Epub 2018/06/06. 10.1016/j.ajpath.2018.04.016 29870741 PMC6099333

[59] Wang H, Song S, Kou G, Li B, Zhang D, Hou S, et al Treatment of hepatocellular carcinoma in a mouse xenograft model with an immunotoxin which is engineered to eliminate vascular leak syndrome. Cancer Immunol Immunother. 2007;56(11):1775–83. Epub 2007/04/14. 10.1007/s00262-007-0321-4 . 17431617 PMC11030707

[60] Weldon JE, Xiang L, Zhang J, Beers R, Walker DA, Onda M, et al A recombinant immunotoxin against the tumor-associated antigen mesothelin reengineered for high activity, low off-target toxicity, and reduced antigenicity. Mol Cancer Ther. 2013;12(1):48–57. Epub 2012/11/09. 10.1158/1535-7163.MCT-12-0336 23136186 PMC3546136

[61] Bauss F, Lechmann M, Krippendorff B-F, Staack R, Herting F, Festag M, et al Characterization of a re-engineered, mesothelin-targeted *Pseudomonas* exotoxin fusion protein for lung cancer therapy. Mol Oncol. 2016;10(8):1317–29. Epub 2016/08/11. 10.1016/j.molonc.2016.07.003 27507537 PMC5423209

