# Supplementary figures and images for: An immunotoxin targeting Ebola virus glycoprotein inhibits Ebola virus production from infected cells
Source: PLoS One. 2021 Jan 7;16(1):e0245024. doi: 10.1371/journal.pone.0245024 (PMC7790382; doi:10.1371/journal.pone.0245024)

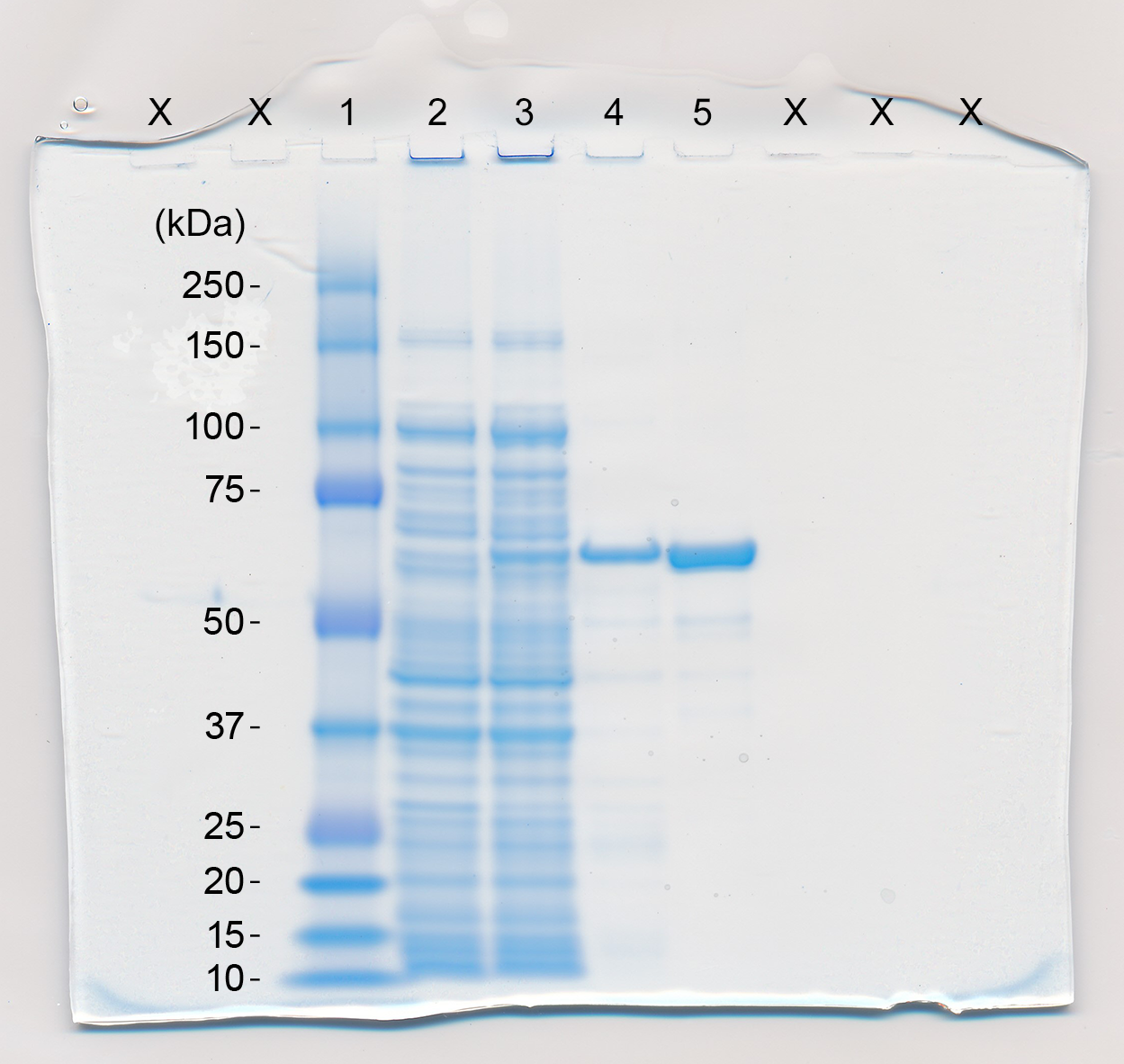

Supplement: S1 Raw image — (TIF) [file pone.0245024.s001.tif]
